# Maternal Microchimerism: Increased in the Insulin Positive Compartment of Type 1 Diabetes Pancreas but Not in Infiltrating Immune Cells or Replicating Islet Cells

**DOI:** 10.1371/journal.pone.0086985

**Published:** 2014-01-31

**Authors:** Jody Ye, Marta Vives-Pi, Kathleen M. Gillespie

**Affiliations:** 1 Diabetes and Metabolism Unit, School of Clinical Sciences, University of Bristol, Bristol, United Kingdom; 2 Immunology Department, Institut de Recerca Germans Trias i Pujol, Carretera Canyet s/n, Badalona, Spain; University of Bremen, Germany

## Abstract

**Background:**

Maternal microchimeric cells (MMc) transfer across the placenta during pregnancy. Increased levels of MMc have been observed in several autoimmune diseases including type 1 diabetes but their role is unknown. It has been suggested that MMc are 1) effector cells of the immune response, 2) targets of the autoimmune response or 3) play a role in tissue repair. The aim of this study was to define the cellular phenotype of MMc in control (n = 14) and type 1 diabetes pancreas (n = 8).

**Methods:**

Using sex chromosome-based fluorescence in-situ hybridization, MMc were identified in male pancreas and their phenotype determined by concomitant immunofluorescence.

**Results:**

In normal pancreas, MMc positive for endocrine, exocrine, duct and acinar markers were identified suggesting that these cells are derived from maternal progenitors. Increased frequencies of MMc were observed in type 1 diabetes pancreas (p = 0.03) with particular enrichment in the insulin positive fraction (p = 0.01). MMc did not contribute to infiltrating immune cells or Ki67+ islet cell populations in type 1 diabetes.

**Conclusion:**

These studies provide support for the hypothesis that MMc in human pancreas are derived from pancreatic precursors. Increased frequencies of MMc beta cells may contribute to the initiation of autoimmunity or to tissue repair but do not infiltrate islets in type 1 diabetes.

## Introduction

It is generally accepted that islet autoimmunity results from loss of tolerance to pancreatic self-peptides very early in life but despite decades of research, the origins of autoimmunity remain obscure. Birth cohort studies agree that seroconversion to insulin autoantibody positivity occurs from the age of 6 months onwards [1] raising the possibility that pre- or neonatal events may influence autoimmunity. The transfer of cells from mother to child in pregnancy represents the earliest immunological insult for the developing fetus. Maternal cells induce fetal CD4+CD25highFoxP3+Tregs that suppress anti-maternal immunity with effects that remain detectable into adulthood [2]. This suggests the active maintenance of immune tolerance to genetically distinct maternal cells in health and is supported by the observation of improved survival of renal transplants from sibling donors who share the non-inherited maternal HLA with the recipient [3].

Over the last decade, elevated levels of maternal cells have been reported in several autoimmune diseases (reviewed in [4], [5]) with several different hypotheses proposed; some suggest that MMc are effector cells of the immune response [6], others that semi-allogeneic MMc in specific host tissues may act as triggers of autoimmunity [7] while it has also been postulated that some MMc, like fetal microchimeric cells, may play a role in regeneration of damaged tissues [8]. We previously observed increased levels of MMc in the pancreas and periphery of individuals with type 1 diabetes [9], [10]. Phenotyping of MMc was limited however because of difficulties combining FISH and immunofluorescence on historical autopsy samples as well as availability of well-matched controls.

In this study, we determined the frequency and phenotypes of MMc in pancreatic autopsy sections from normal individuals, as well as in males with type 1 diabetes and age-matched controls.

## Materials and Methods

### Ethics statement

Full written informed consent was available for all samples tested and appropriate local ethical approval was obtained from Southmead Research Ethics Committee (Ref:04/Q2002/35:The role of maternal microchimerism in type 1 diabetes). The samples used in this study were obtained from the following tissue banks: Network for pancreatic orgon donors with diabetes (nPOD - http://www.jdrfnpod.org/), the Glasgow Tissue Bank (http://www.nhsggc.org.uk/content/default.asp?page=s1685) and the Erasmus Tissue Bank (http://www.erasmusmc.nl/pathologie/clinicalpathology/tissuebank)”.

### Human tissues to phenotype MMc in control pancreas

To determine phenotype of MMc in normal human pancreas, 4 µm sections of 9 formalin fixed and paraffin embedded (FFPE) control male human pancreases provided by Dr. Ronald de Krijger, Erasmus University, Rotterdam, The Netherlands, were examined (Table 1, controls 6 to 14). These tissues were considered normal on pathological examination and no history was available on twin pregnancies or transfusion history. Female tissues were excluded because the detection of maternal cells was based on sex-chromosome discrimination.

**Table 1:** Age, duration of diabetes and inflammatory status in pancreases from individuals with type 1 diabetes, 5 age matched controls for T1D cases, and 9 control pancreases.

	nPOD Case ID	Age	Duration of diabetes	Insulin positivity	Beta cell destruction status
**T1D case 1**	6051	20.3 years	13 years	Yes	Mild infiltration, partial beta cell destruction
**T1D case 2**	6084	14.2 years	4 years	Yes	Mild to heavy infiltration, partial beta cell destruction
**T1D case 3**	6069	22.6 years	7 years	Yes	Mild infiltration, partial beta cell destruction
**T1D case 4**	6180	27.1 years	11years	No	Complete beta cell destruction
**T1D case 5**	6081	31.4 years	15 years	Yes	Partial beta cell destruction
**T1D case 6**	6088	31.2 years	5 years	Yes	Partial beta cell destruction
**T1D case 7**	-	15 years	6 months	Yes	Partial beta cell destruction
**T1D case 8**	-	17 years	Recent onset	Yes	Partial beta cell destruction
**Control 1 for T1D case 1**	6174	20.8 years	-	Yes	-
**Control 2 for T1D case 2**	6099	14.2 years	-	Yes	-
**Control 3 for T1D case 3**	6162	22.7 years	-	Yes	-
**Control 4 for T1D case 4**	6055	27 years	-	Yes	-
**Control 5 (for T1D cases 5 and 6)**	6030	30.1 years	-	Yes	-
**Control 6**	-	36–40w gestational	-	Yes	-
**Control 7**	-	37w gestational	-	Yes	-
**Control 8**	-	40w2days gestational	-	Yes	-
**Control 9**	-	6 weeks	-	Yes	-
**Control 10**	-	21 months	-	Yes	-
**Control 11**	-	3 years	-	Yes	-
**Control 12**	-	4 years	-	Yes	-
**Control 13**	-	11 years	-	Yes	-
**Control 14**	-	13 years	-	Yes	-

### T1D and age matched normal human pancreas

4 µm sections of FFPE male human pancreases from 6 long standing T1D patients (T1D cases 1 to 6) and 5 aged matched controls were obtained from the Network for Pancreatic Organ Donors (nPOD) and are listed with nPOD IDs in Table 1. Two recent onset T1D pancreases (T1D cases 7 and 8) were provided by Dr Alan Foulis, Glasgow Royal Infirmary, Glasgow, UK.

### Fresh human islets

Human islets with estimated 5–10,000 IEQ, purity 70–80% isolated from a 59-year-old male cadaveric donor were obtained from the Islet Isolation Centre, University of Oxford, Oxford, UK. Individual islets were cultured on type I collagen coated coverslips in CMRL1066 basal medium with 10% fetal calf serum (FCS), 2 mM L-glutamine, 100 units/ml penicillin and 100 µg/ml streptomycin at 37°C for FISH and immunofluorescence.

### Concomitant fluorescence in situ hybridization (FISH) and immunofluorescence

Paraffin sections were deparaffinized in a xylene series followed by heat mediated antigen retrieval. Slides were then dehydrated in an ethanol series (70%, 80% and 100%) and air dried. Frozen sections were fixed in ice-cold pure ethanol for 10 min and air-dried prior to *in situ* hybridization. Purified human islets were cultured on rat tail type I collagen coated coverslips. They were fixed in 1% paraformaldehyde for 15 min, then simultaneously permeabilized and blocked in 0.1% (w/v) saponin (Sigma, S4521), 3% BSA solution for 45 min, and subsequently dehydrated in ethanol series. After pre-treatments, tissue sections or cell preparations were incubated with X/Y chromosome FISH probes (Vysis CEP X Spectrum Orange™/Y Spectrum Green™ Direct labelled Fluorescent DNA probe, Abbot Molecular Inc., Des Plaines, IL) as described previously [10]. To study aneuploidy/polyploidy, the CEP 18 probe (Abbot Molecular Inc.) was used in combination with the X/Y probe. After post-hybridization washes, sections or cells were incubated with primary antibodies (and controls as appropriate) specific for insulin (DAKO, A0564), glucagon (DAKO, A0565), somatostatin (Santa Cruz, SC-13099), GATA4 (Santa Cruz, SC-1237), Cytokeratin-19 (Abcam, Ab9221), Ki67 (Abcam, Ab833), CD68 (DAKO, m0876), CD45 (DAKO, M0701), nestin (Millipore MAB5326), or CD34 (Invitrogen, 073403) for 2 hrs at 37°C. After probing with the corresponding fluorescent secondary antibody for 1 hr at room temperature, sections were washed three times in 1× PBS solution, dehydrated, and counterstained with DAPI permanent mounting media (VECTASHIELD®, Vector laboratories, CA).

### Image analysis

#### Fluorescent imaging

Images were captured using an Olympus BX41 fluorescent microscope fitted with an AxioCam MRm digital camera (Zeiss, Germany). The X and Y chromosomes were analyzed using the ISIS fluorescent imaging software (Zeiss, Germany) designed for FISH.

#### Confocal microscopy

To confirm MMc frequencies, pancreatic tissue sections were screened using a Leica SP5 confocal imaging system at the Wolfson Bioimaging Facility, School of Biochemistry, University of Bristol, UK. Z-stack images of each tissue section were analyzed for FISH and immunofluorescence to allow visualization of X and Y chromosome signals in all planes of the nucleus.

### Counting strategy

FISH signals were checked in individual filter channels to ensure signal fidelity and that the visualization of two red dots representing two copies of the X chromosome was not caused by immunofluorescence cross talk. Only cells that showed clear XY (red and green dot) or XX (two red dots) signals within single nuclei were counted. Any cells with overlapping nuclei were excluded from the analysis. X chromosome “splits” that occasionally appeared as two juxtapositional dots due to DNA breakage were not considered as XX signals. A FISH success rate (the frequency of nuclei with two clear signals visible) of greater than 60% was required before further analysis.

All T1D and matched control tissues were stained for FISH and insulin. Overall, as many nuclei as possible were counted (>1000 at least), including at least 20 islets per section. MMc were counted in insulin positive and insulin negative cell fractions. For T1D case 1, 2, and 3, MMc were also examined in CD45+ population. An additional independent, blinded scorer reviewed each candidate female cell.

### Statistical analysis

Differences in MMc frequencies between T1D patients and controls were analysed using two-tailed t tests (Graphpad Prism 5).

## Results

### MMc are endocrine and exocrine cells in normal pancreas

**Figure 1:**
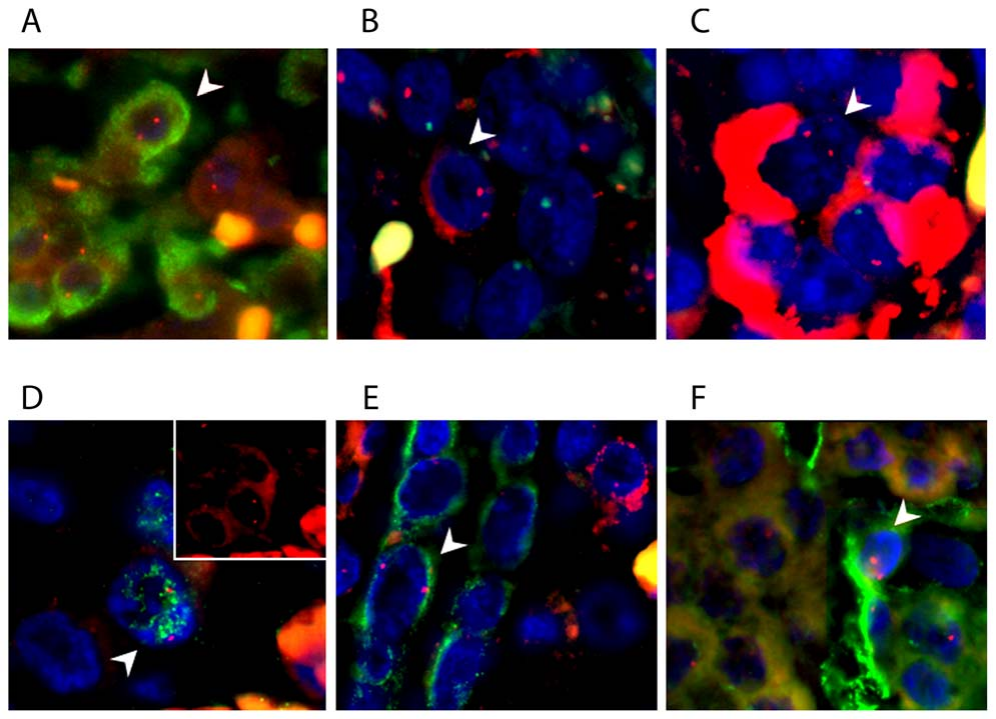
Phenotypes of MMc in male normal human pancreases, the X chromosome is stained as red dot, the Y chromosome is stained as green dot; an MMc is visualized as a nucleus containing two red dots; male cells contain a red and a green dot in their nuclei; panel of images showing an MMc was positive for a) insulin (green, cytoplasmic staining) in an 11 month-old pancreas; b) glucagon (red, cytoplasmic staining) in a 13 year-old pancreas; c) somatostatin (red, cytoplasmic staining) in a 36–40 week fetal pancreas; d) GATA4 (green, nuclear staining) in a 36–40 week fetal pancreas, the upper right image shows the two X chromosomes captured in Spectrum Orange channel; e) cytokeratin 19 (green cytoplasmic staining) in a 36–40 week fetal pancreas; and f) CD34 (green cytoplasmic staining) in a 36–40 week fetal pancreas; nuclei were counterstained with DAPI; magnification 40×.

MMc were identified in all normal pancreas samples tested. Insulin, glucagon, and rarely somatostatin positive MMc were present in the islets of Langerhans, suggesting that they were beta cells, alpha cells, and delta cells of normal endocrine compartments (Figure 1 a–c). MMc were also identified outside the islets. To determine whether they were acinar cells, the expression of GATA4, a transcription factor specific for acinar cells in pancreas was examined [11] [12] and GATA4 positive MMc were observed (Figure 1 d). In the ductal epithelium, rare cytokeratin-19 positive MMc were detected. Co-staining for cytokeratin-19 and insulin demonstrated occasional insulin positive cells close to the duct but these were not MMc (Figure 1 e). Additionally, rare CD34+ MMc were scattered in the strands of microvasculature (Figure 1 f), co-stained for VE-cadherin (data not shown) suggesting that these cells are endothelial in origin.

### MMc in fresh human islets

**Figure 2:**
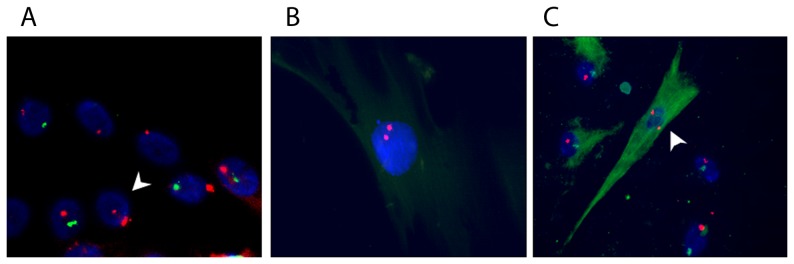
MMc were present in isolated male human islets; a) X/Y FISH demonstrating an MMc containing two X chromosomes (arrow indicated) in freshly isolated male islet cells; b) an MMc identified at passage 5 in male human islet culture; c) an MMc was expressing nestin (green); nuclei were counterstained with DAPI, magnification 40×.

MMc were demonstrated in intact human islets cultured for 7 days and in islet-derived cells cultured for 43 days at passage 5 (Figure 2 a and b). Concomitant immunolabeling showed an MMc positive for nestin (Figure 2 c), an intermediate filament that is transiently expressed by islet cells that undergo dedifferentiation. This implies that *in vitro*, MMc propagate and dedifferentiate as host cells do [13], [14].

### MMc verification: confocal microscopy, aneuploidy/polyploidy and phagocytosis

XY chromosome was combined with chromosome 18 FISH determine whether cells identified as MMc could result from aneuploidy/polyploidy. A typical MMc contained XX, 1818 signals (Figure S1), indicating the MMc are diploid, not polyploid. Finally, it is possible that XX cells in T1D islets could result from phagocytosis but again co-staining MMc with macrophage marker proved that this was not the case (Figure S2).

### Fetal MMc are capable of replicating

**Figure 3:**
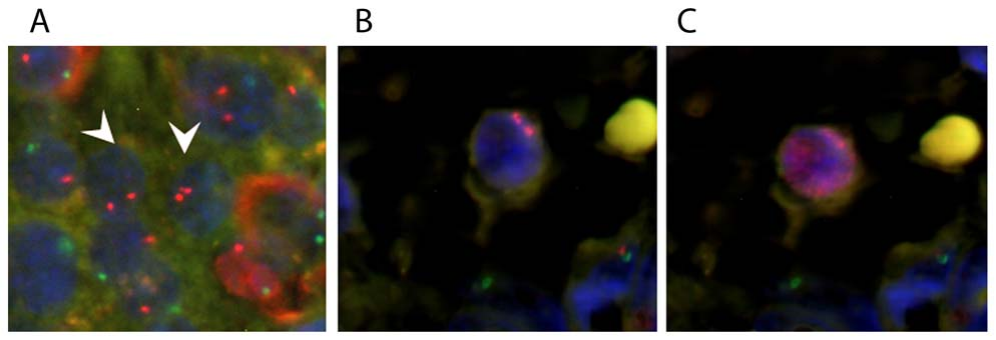
MMc can replicate in fetal pancreas; a) two MMc located in close proximity; b) an MMc in a 37-week gestational pancreas; and c) this cell is positive for Ki67 (red, nuclear staining); nuclei were counterstained with DAPI; magnification 40× for Figure 3a, and 100× for Figure 3b and 3c.

Some MMc were present in close proximity (Figure 3 a). Given the extremely low frequencies of MMc, we questioned whether some MMc were able to proliferate. In agreement with this, an MMc positive for the replication marker, Ki67 was identified in exocrine tissue from a normal 37-week gestational pancreas, suggesting that these cells are capable of undergoing mitosis during development (Figure 3 b and c).

### MMc levels are higher in T1D pancreas overall, and in the insulin positive compartment, compared with age-matched controls

**Figure 4:**
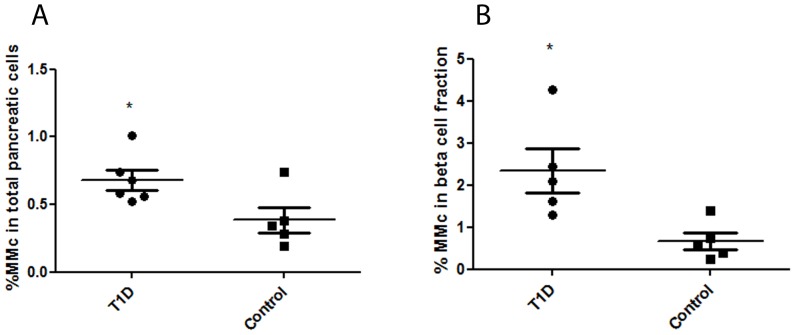
Frequencies of MMc in male T1D and control pancreases a) frequencies of total pancreatic MMc in T1D patients (n = 6) vs. age-matched controls (n = 5) p = 0.03; b) frequencies of MMc in insulin positive cell fractions in T1D patients (n = 5) vs. age-matched controls (n = 5), p = 0.0166.

As shown in Figure 4 a, the frequencies of total pancreatic MMc were significantly higher in T1D (0.68±0.07%, n = 6) compared with control pancreas (0.39±0.09%, n = 5) (two tailed t test, p = 0.033). Analysis of insulin positive MMc revealed enrichment of maternal cells among the beta cell fraction in the islets of T1D pancreases (2.34±0.5%, n = 5 in T1D samples vs. 0.66±0.2%, n = 5 in control samples) (two tailed t test, p = 0.0166) (Figure 4b). Nuclei containing XXY and/or XYY aneuploidy were observed at similar rates in T1D and control pancreas (0.6±0.1% in T1D and 0.64±0.3% in controls) (Table 2).

**Table 2:** Frequencies of MMc in T1D and control male human pancreases.

	Total cells	Total No. MMc/(%)	No. of insulin^+^ MMc/(%)	Anueploidy/Polyploidy (%)
T1D case 1	2865	29/1.01%	12/4.27%	1.08%
T1D case 2	2417	18/0.74%	11/2.44%	0.78%
T1D case 3	2956	17/0.58%	10/1.62%	0.74%
T1D case 4	2666	18/0.68%	6/1.30%	0.15%
T1D case 5	2321	12/0.52%	0/0%	0.47%
T1D case 6	2330	13/0.56%	7/2.09%	0.43%
Control 1	2130	6/0.28%	2/0.53%	1.88%
Control 2	1577	6/0.38%	2/0.38%	0.45%
Control 3	1483	11/0.74%	7/1.4%	0.27%
Control 4	1571	3/0.19%	1/0.24%	0.45%
Control 5	2620	9/0.34%	7/0.75%	0.15%

### MMc do not infiltrate the islets in autoimmune diabetes

To assess whether maternal cells are present in insulitis, two recent onset T1D pancreases, three long-standing T1D pancreases, and three age-matched control pancreases underwent X/Y FISH and CD45 staining. In all recent onset and long standing pancreases examined, >1400 cells were scored per sample. CD45+ frequencies varied from 1–20% in T1D pancreas. All CD45+ leukocytes observed contained X and Y chromosomes (Figure 6a and Table S1). In age-matched control male pancreases, CD45+ cells were mostly located in the vasculature (frequencies between 0.2–2.06%) and none was female.

### Replicating cells in T1D pancreas are not MMc

**Figure 5:**
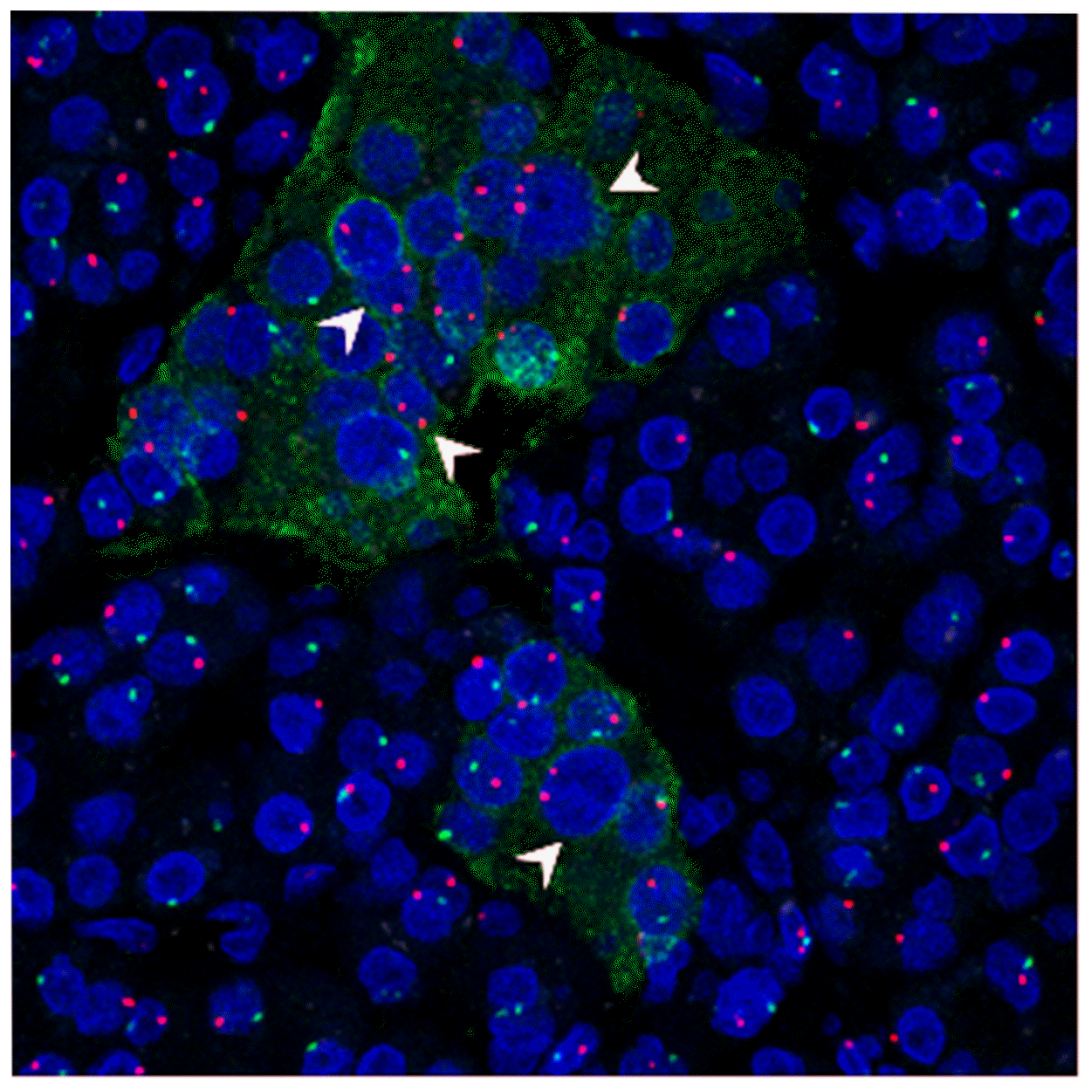
MMc in a 14 year old boy who had type 1 diabetes for 4 years (nPOD case ID: 6084). X chromosome is stained as red dot, Y chromosome is stained as green dot; a) insulin (green, cytoplasmic staining); nuclei were counterstained with DAPI; magnification 40×.

**Figure 6:**
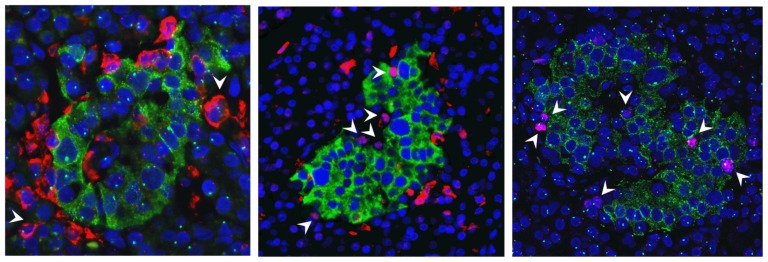
CD45+ leukocytes and Ki67+ proliferating endocrine cells were host derived in the pancreases with T1D; a) X/Y FISH, insulin (green), and CD45 (red) immunofluorescence; arrow indicates a CD45+ male cell b) insulin (green), CD45 (red) and Ki67 (pink, nuclear staining) immunofluorescence; arrows indicate Ki67+ proliferating cells that were CD45 negative; c) X/Y FISH, Ki67 (pink) and insulin (green); arrows indicate Ki67+ proliferating male cells; nuclei were counterstained with DAPI.

As shown on Figure 5, MMc are over-represented in some T1D islets. To determine whether these MMc could be replicating cells, T1D and control sections with intact islets were co-stained with Ki67, insulin and CD45. In T1D case 2, Ki67 positive replicating cells were observed in both endocrine and exocrine tissue but proliferating endocrine cells were not present in an age-matched control (1.09% of 8055 cells examined compared with 0.15% of 6688 cells examined). Proliferating lymphocytes were excluded by CD45 co-staining (Figure 6 b). A total of 27 islets were examined in the T1D tissue, 59 endocrine cells were Ki67 immunopositive and 12 (20%) were dual positive for insulin. X/Y FISH analysis showed that all Ki67 positive cells were male (Figure 6 c). In age matched control tissue, 29 islets were examined and no replicating endocrine cells were detected. 10 scattered Ki67+ cells were found in the acini, which were all male cells.

## Discussion

The phenomenon of bi-directional cell trafficking across the placental barrier is well established but the biological consequences remain elusive. Chimeric cells of maternal origin reside in the periphery and in multiple somatic tissues. Previously, we detected the presence of MMc in male human pancreas and increased levels in type 1 diabetic pancreas [9], [10]. Here, we demonstrate that MMc exist in all pancreatic cell subsets including the endocrine, exocrine, and vascular endothelial lineages, implying that they are likely to be terminally differentiated cells derived from maternal multi/pluripotent progenitors and suggesting maternal stem cell differentiation in the developing pancreas. The frequency of MMc is increased in type 1 diabetes pancreases with particular enrichment in beta cells. This enrichment was not due to infiltrating immune cells or to islet cell replication.

A strength of this study was the availability of high quality type 1 diabetes and control tissue for analysis and specific methodologies to detect and phenotype cells with two X chromosomes in male tissue. There are also some limitations: insulin is a good but not perfect marker of beta cell phenotype as islets are cell dense and cytoplasmic staining can be difficult to assign to a given nucleus. Analysing maternal cell frequency by counting several thousand cells per section using confocal imaging is labour intensive and although each MMc has been confirmed by an independent second scorer, a degree of subjectivity remains. Similarly, polyploid beta cells, which have previously been described in human pancreas [15], could be potentially erroneously classified as XX signals. XY chromosome FISH was therefore combined with chromosome 18 FISH to determine whether 4 copies of chromosome 18 could be identified in “MMc”. These experiments did not identify a single miscalled MMc. Finally, it is possible that XX cells in T1D islets could result from phagocytosis but again our data showed that this was not the case. Unequivocal identification of the ‘maternal origin’ of MMc by single cell laser capture and DNA profiling are under development and would eliminate these queries in future studies. Our analysis focused on longstanding diabetes and we found higher frequencies of insulin positive MMc compared with the recent onset case reported previously by Nelson and colleagues [9]. While this could suggest that MMc frequency increases after diagnosis or that MMc are resistant to lysis, further larger studies taking genetic background into consideration are required to address these questions.

These studies raise many questions regarding the role of the increased frequency of maternal cells in type 1 diabetes. While we have shown that MMc are not infiltrating cells in T1D islets, they could potentially be involved in early events in type 1 diabetes initiation or in the response to islet autoimmunity. Analysis of MMc pancreatic sections from islet autoantibody positive individuals who have not yet developed type 1 diabetes will be required to shed light on this conundrum. Elevated levels of semi-allogeneic maternal cells could potentially trigger the loss of tolerance to self-antigens in autoimmunity. The importance of early life events in susceptibility to type 1 diabetes is increasingly clear [16] and maternal cells have been shown to shape fetal immune development [2]. In the case of autoimmunity, it is unclear whether there is a threshold of MMc level at certain times during fetal immune development that tightly regulates immune balance and whether levels above the threshold could lead to the breakdown of anti-maternal tolerance. The classic model of type 1 diabetes involves initial death of a few beta cells, followed by cascades of epitope spreading and beta cell destruction [17]. Birth cohort studies demonstrate an explosion of islet autoimmunity between 6 months and 3 years of age [18] [19] when islet reorganization is occurring. If loss of maternal immune tolerance occurs, exposure to maternal beta cell alloantigens upon either physiological stress (i.e. beta cell apoptosis during islet remodelling) [20]–[22] or environmental insult could trigger diabetes. It remains to be addressed that whether there are interactions between maternal cells/antigens with host immune cells in the para-pancreatic lymph nodes which may modulate islet autoimmunity. Direct presentation of host beta cell antigens by maternal antigen presenting cells (APC) or indirect presentation of maternal beta cell antigens by host APC may trigger autoimmunity but these hypotheses need to be tested in animal models.

Recent data from a mouse model suggests that MMc could initiate autoimmunity by recognizing fetal antigens. Roy et al. observed that male litters exposed to fetal beta cell specific maternal T cells were predisposed to autoimmune diabetes, but the islet infiltrating lymphocytes were host derived, as we have observed in human type 1 diabetes. They further speculated that maternal T cells may have modified fetal antigen presenting cells towards a more immunogenic self-reactive phenotype [23].

Alternatively, the wide tissue distribution of MMc and the presence of maternal stem cell pools in non-lymphoid tissues [24] led to the suggestion that MMc may participate in tissue regeneration. Whether increased frequencies of MMc result from maternal stem cells contributing to attempted tissue regeneration remains to be proven. Bonner-Weir et al. suggested that ductal cells may transdifferentiate into new islet beta cells [25]. We identified MMc in ductal cells and observed adjacent insulin positive cells but these insulin positive cells were not MMc.

In conclusion, MMc in human pancreas exist in multiple cell subsets. Levels of MMc are increased in type 1 diabetes pancreas with highest levels in T1D islets. This could potentially trigger the loss of anti-maternal immune tolerance and maternal beta cell antigens may serve as immune targets but MMc are not infiltrating immune cells in type 1 diabetes.

## Supporting Information

Figure S1 **A typical MMc in a 14 year-old normal male human pancreas contains two copies of the X chromosome and chromosome 18.** The X chromosome is labelled as red dot (Spectrum Orange), the Y chromosome is labelled as green dot (Spectrum Orange), and chromosome 18 is labelled as yellow-orange dot (pseudo-colour) (Spectrum Aqua); nuclei were counterstained with DAPI. a–c) represents different focal planes generated in confocal Z-stack scan, d) represents merged image. Magnification 63×.
(PDF)

Figure S2 **MMc signals are not artefacts resulting from phagocytosis; a) an insulin positive (Texas Red) MMc in a 3 year old normal pancreas is present independent of b) a male CD68 positive macrophage; images are taken at 100× magnification.**
(PDF)

Table S1 **Number and frequencies of CD45+ cells examined for X/Y FISH in T1D and control human pancreases.**
(DOCX)

## References

[1] Ziegler AG, Bonifacio E (2012) Group B-BS (2012) Age-related islet autoantibody incidence in offspring of patients with type 1 diabetes. Diabetologia 55: 1937–1943. 22289814 10.1007/s00125-012-2472-x

[2] Mold JE, Michaelsson J, Burt TD, Muench MO, Beckerman KP, et al (2008) Maternal alloantigens promote the development of tolerogenic fetal regulatory T cells in utero. Science 322: 1562–1565. 19056990 10.1126/science.1164511 PMC2648820

[3] Burlingham WJ, Grailer AP, Heisey DM, Claas FH, Norman D, et al (1998) The effect of tolerance to noninherited maternal HLA antigens on the survival of renal transplants from sibling donors. The New England journal of medicine 339: 1657–1664. 9834302 10.1056/NEJM199812033392302

[4] Nelson JL (2008) Your cells are my cells. Sci Am 298: 64–71. 18376674

[5] Nelson JL (2012) The otherness of self: microchimerism in health and disease. Trends Immunol 33: 421–7. 22609148 10.1016/j.it.2012.03.002 PMC3516290

[6] Reed AM, McNallan K, Wettstein P, Vehe R, Ober C (2004) Does HLA-dependent chimerism underlie the pathogenesis of juvenile dermatomyositis? Journal of immunology 172: 5041–5046. 10.4049/jimmunol.172.8.5041 15067086

[7] Stevens AM, Hermes HM, Rutledge JC, Buyon JP, Nelson JL (2003) Myocardial-tissue-specific phenotype of maternal microchimerism in neonatal lupus congenital heart block. Lancet 362: 1617–1623. 14630442 10.1016/S0140-6736(03)14795-2

[8] O'Donoghue K, Chan J, de la Fuente J, Kennea N, Sandison A, et al (2004) Microchimerism in female bone marrow and bone decades after fetal mesenchymal stem-cell trafficking in pregnancy. Lancet 364: 179–182. 15246731 10.1016/S0140-6736(04)16631-2

[9] Nelson JL, Gillespie KM, Lambert NC, Stevens AM, Loubiere LS, et al (2007) Maternal microchimerism in peripheral blood in type 1 diabetes and pancreatic islet beta cell microchimerism. Proc Natl Acad Sci U S A 104: 1637–1642. 17244711 10.1073/pnas.0606169104 PMC1785262

[10] Vanzyl B, Planas R, Ye Y, Foulis A, de Krijger RR, et al (2010) Why are levels of maternal microchimerism higher in type 1 diabetes pancreas? Chimerism 1: 45–50. 21327046 10.4161/chim.1.2.13891 PMC3023622

[11] Ketola I, Otonkoski T, Pulkkinen MA, Niemi H, Palgi J, et al (2004) Transcription factor GATA-6 is expressed in the endocrine and GATA-4 in the exocrine pancreas. Mol Cell Endocrinol 226: 51–57. 15489005 10.1016/j.mce.2004.06.007

[12] Karafin MS, Cummings CT, Fu B, Iacobuzio-Donahue CA (2009) The developmental transcription factor Gata4 is overexpressed in pancreatic ductal adenocarcinoma. Int J Clin Exp Pathol 3: 47–55. 19918328 PMC2776266

[13] Gallo R, Gambelli F, Gava B, Sasdelli F, Tellone V, et al (2007) Generation and expansion of multipotent mesenchymal progenitor cells from cultured human pancreatic islets. Cell Death Differ 14: 1860–1871. 17612586 10.1038/sj.cdd.4402199

[14] Russ HA, Ravassard P, Kerr-Conte J, Pattou F, Efrat S (2009) Epithelial-mesenchymal transition in cells expanded in vitro from lineage-traced adult human pancreatic beta cells. PLoS One 4: e6417. 19641613 10.1371/journal.pone.0006417 PMC2712769

[15] Ehrie MG, Swartz FJ (1974) Diploid, tetraploid and octaploid beta cells in the islets of Langerhans of the normal human pancreas. Diabetes 23: 583–588. 4135223 10.2337/diab.23.7.583

[16] Stene LC, Gale EA (2013) The prenatal environment and type 1 diabetes. Diabetologia 10.1007/s00125-013-2929-6 23657800

[17] van Belle TL, Coppieters KT, von Herrath MG (2011) Type 1 diabetes: etiology, immunology, and therapeutic strategies. Physiol Rev 91: 79–118. 21248163 10.1152/physrev.00003.2010

[18] Ziegler AG, Bonifacio E, Grp B-BS (2012) Age-related islet autoantibody incidence in offspring of patients with type 1 diabetes. Diabetologia 55: 1937–1943. 22289814 10.1007/s00125-012-2472-x

[19] Parikka V, Nanto-Salonen K, Saarinen M, Simell T, Ilonen J, et al (2012) Early seroconversion and rapidly increasing autoantibody concentrations predict prepubertal manifestation of type 1 diabetes in children at genetic risk. Diabetologia 55: 1926–1936. 22441569 10.1007/s00125-012-2523-3

[20] Trudeau JD, Dutz JP, Arany E, Hill DJ, Fieldus WE, et al (2000) Neonatal beta-cell apoptosis: a trigger for autoimmune diabetes? Diabetes 49: 1–7. 10615942 10.2337/diabetes.49.1.1

[21] Meier JJ, Butler AE, Saisho Y, Monchamp T, Galasso R, et al (2008) Beta-cell replication is the primary mechanism subserving the postnatal expansion of beta-cell mass in humans. Diabetes 57: 1584–1594. 18334605 10.2337/db07-1369 PMC3697779

[22] Turley S, Poirot L, Hattori M, Benoist C, Mathis D (2003) Physiological beta cell death triggers priming of self-reactive T cells by dendritic cells in a type-1 diabetes model. J Exp Med 198: 1527–1537. 14623908 10.1084/jem.20030966 PMC2194112

[23] Roy E, Leduc M, Guegan S, Rachdi L, Kluger N, et al (2011) Specific maternal microchimeric T cells targeting fetal antigens in beta cells predispose to auto-immune diabetes in the child. J Autoimmun 36: 253–262. 21414756 10.1016/j.jaut.2011.02.003

[24] Dutta P, Burlingham WJ (2010) Stem cell microchimerism and tolerance to non-inherited maternal antigens. Chimerism 1: 2–10. 21132055 10.4161/chim.1.1.12667 PMC2995256

[25] Bonner-Weir S, Inada A, Yatoh S, Li WC, Aye T, et al (2008) Transdifferentiation of pancreatic ductal cells to endocrine beta-cells. Biochem Soc Trans 36: 353–356. 18481956 10.1042/BST0360353

