# Supplementary figures and images for: Maternal Microchimerism: Increased in the Insulin Positive Compartment of Type 1 Diabetes Pancreas but Not in Infiltrating Immune Cells or Replicating Islet Cells
Source: PLoS One. 2014 Jan 31;9(1):e86985. doi: 10.1371/journal.pone.0086985 (PMC3909047; doi:10.1371/journal.pone.0086985)

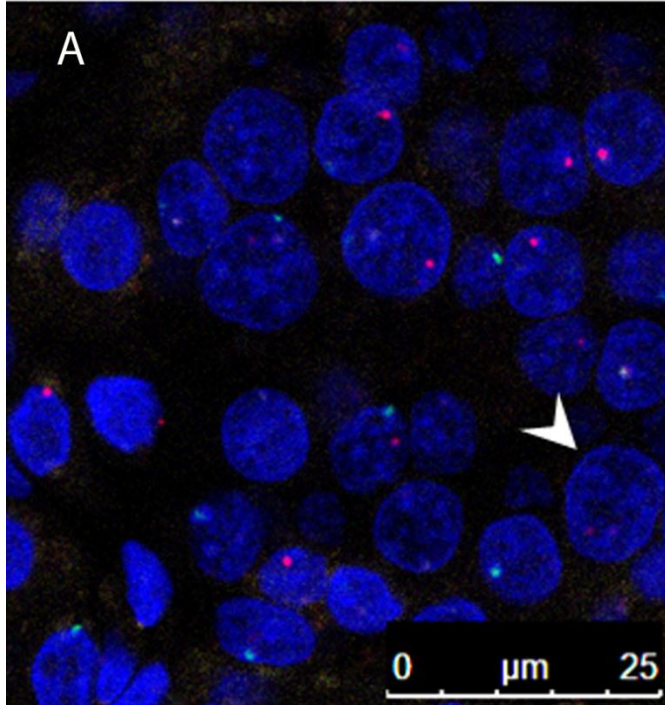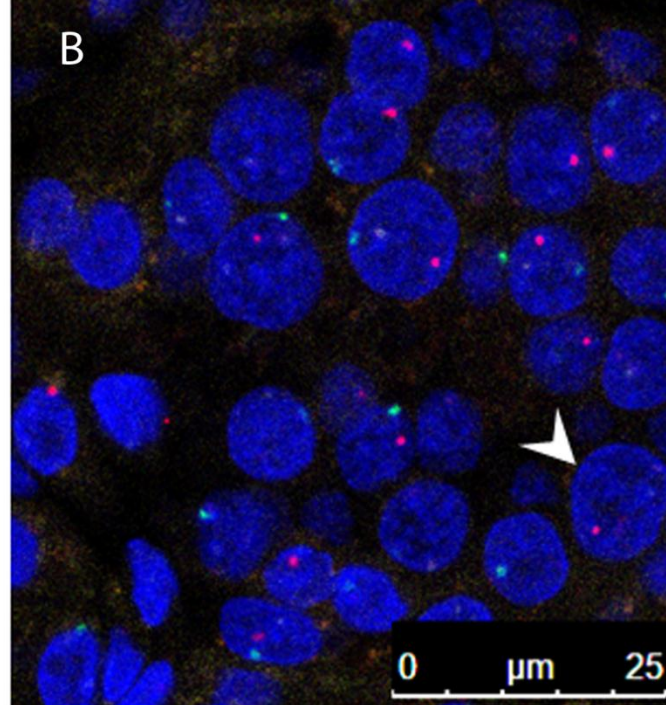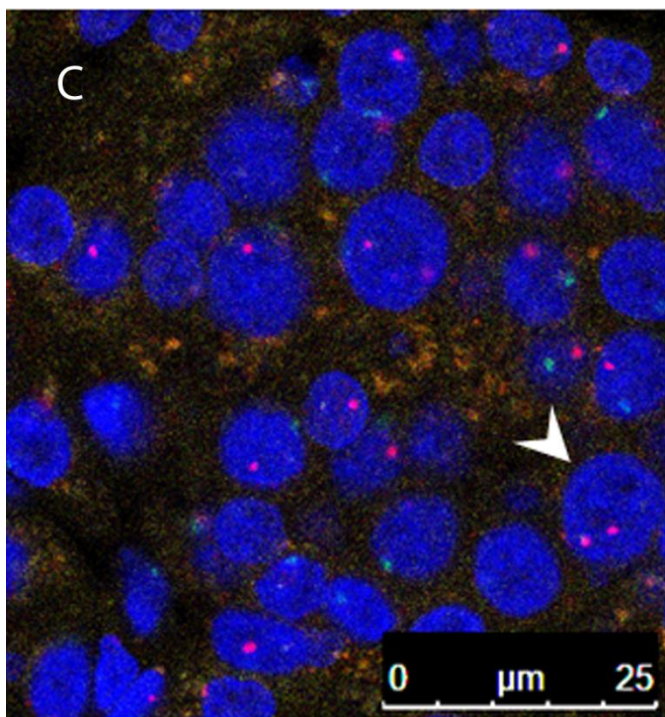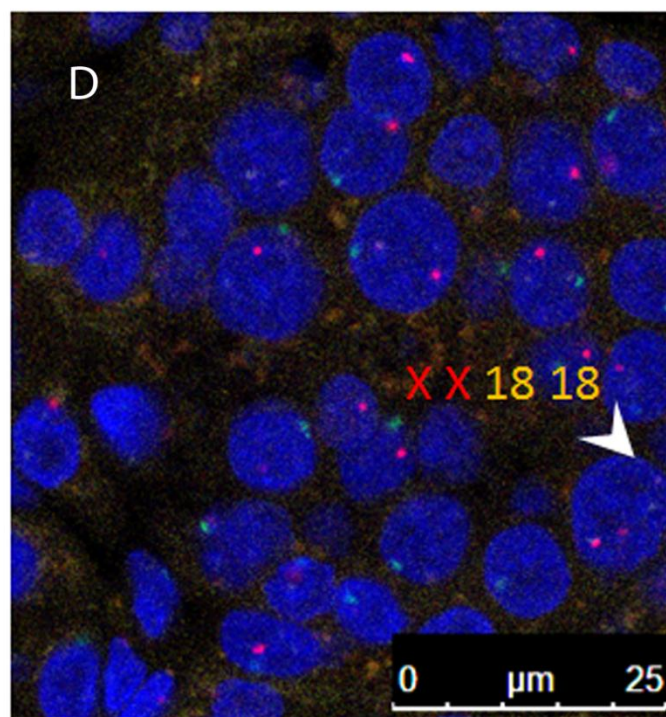

Supplement: Figure S1 — A typical MMc in a 14 year-old normal male human pancreas contains two copies of the X chromosome and chromosome 18. The X chromosome is labelled as red dot (Spectrum Orange), the Y chromosome is labelled as green dot (Spectrum Orange), and chromosome 18 is labelled as yellow-orange dot (pseudo-colour) (Spectrum Aqua); nuclei were counterstained with DAPI. a–c) represents different focal planes generated in confocal Z-stack scan, d) represents merged image. Magnification 63×. (PDF) [file pone.0086985.s001.pdf]

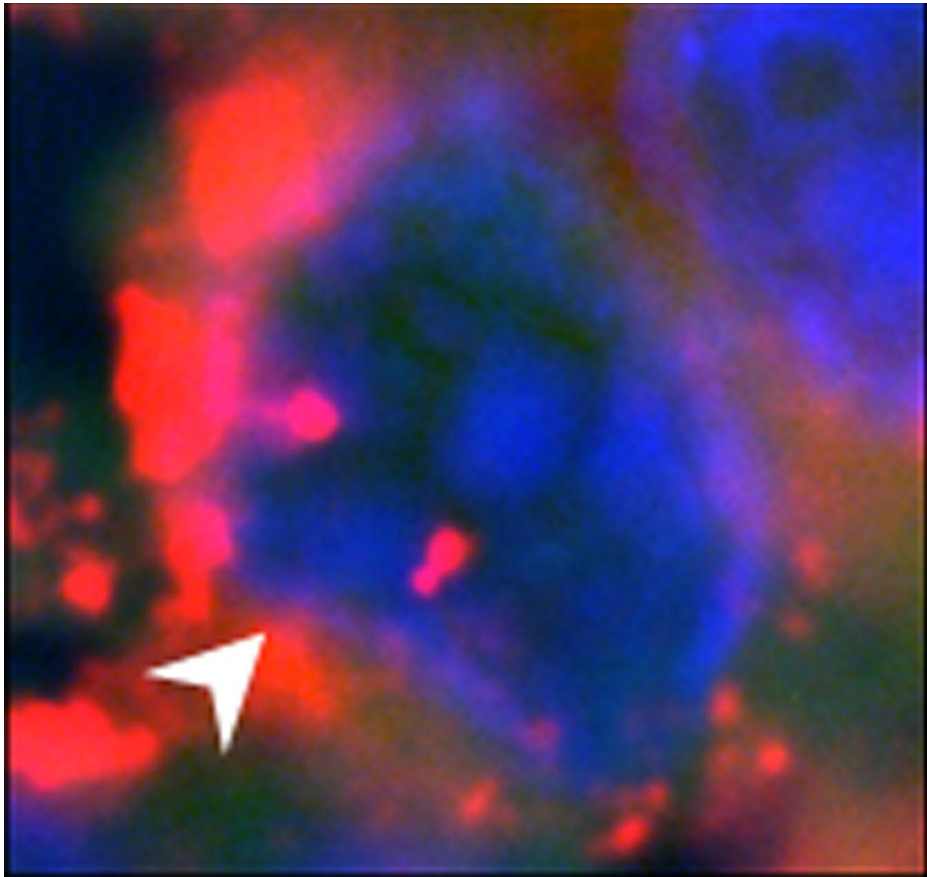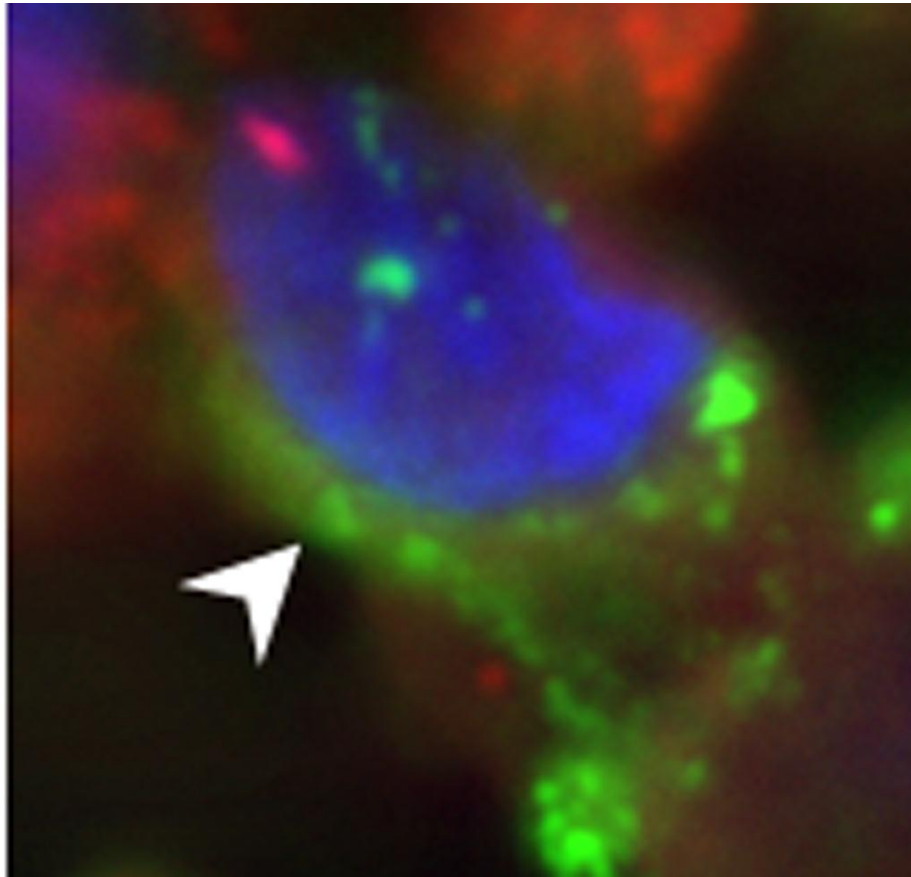

Supplement: Figure S2 — MMc signals are not artefacts resulting from phagocytosis; a) an insulin positive (Texas Red) MMc in a 3 year old normal pancreas is present independent of b) a male CD68 positive macrophage; images are taken at 100× magnification. (PDF) [file pone.0086985.s002.pdf]
