# Supplementary material for: Maternal Microchimerism: Increased in the Insulin Positive Compartment of Type 1 Diabetes Pancreas but Not in Infiltrating Immune Cells or Replicating Islet Cells
Source: PLoS One. 2014 Jan 31;9(1):e86985. doi: 10.1371/journal.pone.0086985 (PMC3909047; doi:10.1371/journal.pone.0086985)
Supplement: Table S1 — Number and frequencies of CD45+ cells examined for X/Y FISH in T1D and control human pancreases. (DOCX) [file pone.0086985.s003.docx]

| T1D samples | Total cells scored | CD45+ cells | CD45 frequencies |
| --- | --- | --- | --- |
| T1D Case 1 | 1497 | 309 | 20.6% |
| T1D Case 2 | 2418 | 86 | 2.9% |
| T1D Case 3 | 2954 | 32 | 1.08% |
| T1D Case 7  Recent onset | 5067 | 78 | 1.54% |
| T1D Case 8  Recent onset | 2795 | 26 | 0.93% |
| Control 1 | 2130 | 44 | 2.06% |
| Control 2 | 1577 | 16 | 1.01% |
| Control 3 | 1483 | 3 | 0.20% |
